# Transforming Growth Factor-β Signaling Plays a Pivotal Role in the Interplay Between Osteosarcoma Cells and Their Microenvironment

**DOI:** 10.3389/fonc.2018.00133

**Published:** 2018-04-30

**Authors:** Franck Verrecchia, Françoise Rédini

**Affiliations:** UMR1238 INSERM, Université de Nantes, PHY-OS, “Bone Sarcomas and Remodeling of Calcified Tissues”, Medical School, Nantes, France

**Keywords:** osteosarcoma, transforming growth factor-β, microenvironment, bone, metastases

## Abstract

Osteosarcomas are the most frequent form of primary bone tumors and mainly affect children, adolescents, and young adults. Despite encouraging progress in therapeutic management, including the advent of multidrug chemotherapy, the survival rates have remained unchanged for more than four decades: 75% at 5 years for localized disease, but two groups of patients are still at high risk: metastatic at diagnosis (overall survival around 40% at 5 years) and/or poor responders to chemotherapy (20% at 5 years). Because these tumors are classified as “complex genomic,” it is extremely difficult to determine the signaling pathways that might be targeted by specific therapies. A hypothesis has thus emerged, stating that the particular microenvironment of these tumors may interfere with the tumor cells that promote chemoresistance and the dissemination of metastases. The stroma is composed of a large number of cell types (immune cells, endothelial cells, mesenchymal stromal cells, etc.) which secrete growth factors, such as transforming growth factor-β (TGF-β), which favors the development of primary tumors and dissemination of metastases by constituting a permissive niche at primary and distant sites. Rather than targeting the tumor cells themselves, which are very heterogeneous in osteosarcoma, the hypothesis is instead to target the key actors secreted in the microenvironment, such as TGF-βs, which play a part in tumor progression. In the last decade, numerous studies have shown that overexpression of TGF-β is a hallmark of many cancers, including primary bone tumors. In this context, TGF-β signaling has emerged as a crucial factor in the cross talk between tumor cells and stroma cells in poor-prognosis cancers. Secretion of TGF-β by tumor cells or stroma cells can effectively act in a paracrine manner to regulate the phenotype and functions of the microenvironment to stimulate protumorigenic microenvironmental changes. TGF-β can thus exert its protumorigenic function in primary bone tumors by promoting angiogenesis, bone remodeling and cell migration, and by inhibiting immunosurveillance. This review focuses on the involvement of TGF-β signaling in primary bone tumor development, and the related therapeutic options that may be possible for these tumors.

## Introduction

Osteosarcoma (OS) is the most common malignant primary bone tumor, occurring above all in children, adolescents, or young adults with a median age of onset of 18 years. These tumors occur commonly in the metaphyseal region of the long bones, developing at sites of rapid bone growth (1). The World Health Organization classification of tumors of the soft tissue and bone defines osteosarcoma as a “malignant, bone-forming tumor, divided into several histological subtypes: chondroblastic, fibroblastic, osteoblastic, telangiectasic, or small cells” (2). Some of these histological forms have distinct molecular and biological behaviors. Most osteosarcomas are “conventional OS” (85%), defined as primary intramedullar high-grade malignant tumors in which neoplastic cells produce immature bone or osteoid tissue.

Current treatment associates surgery with combinational chemotherapy which cures at 5 years approximately 70% of patients with localized disease, with response to preoperative chemotherapy as the strongest predictor of overall survival (3). However, survival for patients with metastatic or relapsed disease has remained unchanged over the past 40 years, with an overall survival rate of about 20% at 5 years (4, 5). At the time of diagnosis, 20% of patients present with detectable lung metastases, but it has been estimated that undetectable metastases are present in 80% of cases (6).

New therapeutic options are therefore needed for this type of tumor.

## Osteosarcoma Microenvironment: Potential Therapeutic Targets

Conventional high-grade osteosarcomas are generally genomically unstable tumors with complex karyotypes (7). Rarity and genomic complexity, as well as intra-tumoral and intertumoral heterogeneity, have presented challenges for the molecular characterization of osteosarcomas. These tumors are characterized by chromosomal instability, with high levels of somatic structural variations and copy number alterations (8). Somatic mutations in both TP53 and RB1 are the most frequently reported (9, 10). Other mutated genes include RecQ protein-like 4, which encodes a RecQ helicase, and RUNX2. Another contributor to genomic instability is alternative lengthening of telomeres, which prevents telomere shortening and induces senescence (11). To date, the search for common molecular therapeutic targets in osteosarcoma has been disappointing. In this context, rather than targeting tumor cells themselves, the hypothesis is to target the key actors secreted in the microenvironment and which play a part in tumor progression.

Irrespective of their origins, tumors are heterogeneous cellular entities whose progression greatly depends on reciprocal interactions between genetically altered neoplastic cells and their non-neoplastic counterparts present in the microenvironment. Tumor bulk is therefore composed of differentiated tumor cells, and by cancer stem cells that are combined and interact with normal cells. The interplay between them regulates the production and biological activity of many soluble factors and extracellular matrix components that allow the growth and maintenance of solid tumors (12). Therefore, the reactive stroma plays a key role in the development and progression of cancer. Osteosarcoma originates in bone where there is a high concentration of mesenchymal progenitors. Tumor-associated stroma mainly consists of two major categories of component: (i) the extracellular matrix, composed of structural proteins such as collagen and elastin, specialized proteins such as fibronectin, and proteoglycans such as hyaluronan; (ii) cellular elements, composed of cells surrounding the tumor tissue that play a part in the stromal response, i.e., bone cells, vasculature and endothelial cells, pericytes, immune cells such as macrophages [tumor-associated macrophages (TAMs)] and lymphocytes, and mesenchymal stromal cells (MSC). In addition, fibroblasts that differentiate from MSC and usually switch to a tumor-promoting cell phenotype, called the cancer-associated fibroblasts, are also present in the tumor microenvironment (TME) (13).

Mesenchymal stromal cells are involved in osteosarcoma growth and progression, through cross-feeding of the tumor cells *via* the release of cytokines and soluble growth factors, by helping in migration, proliferation and stemness, membrane cross-talk *via* microvesicle secretion, metabolic reprogramming of tumor cells, and immune escape. MSC are non-hematopoietic precursors found in the bone marrow. They contribute to the maintenance and regeneration of a variety of tissues of mesodermal lineage, including bone.

**Figure 1:**
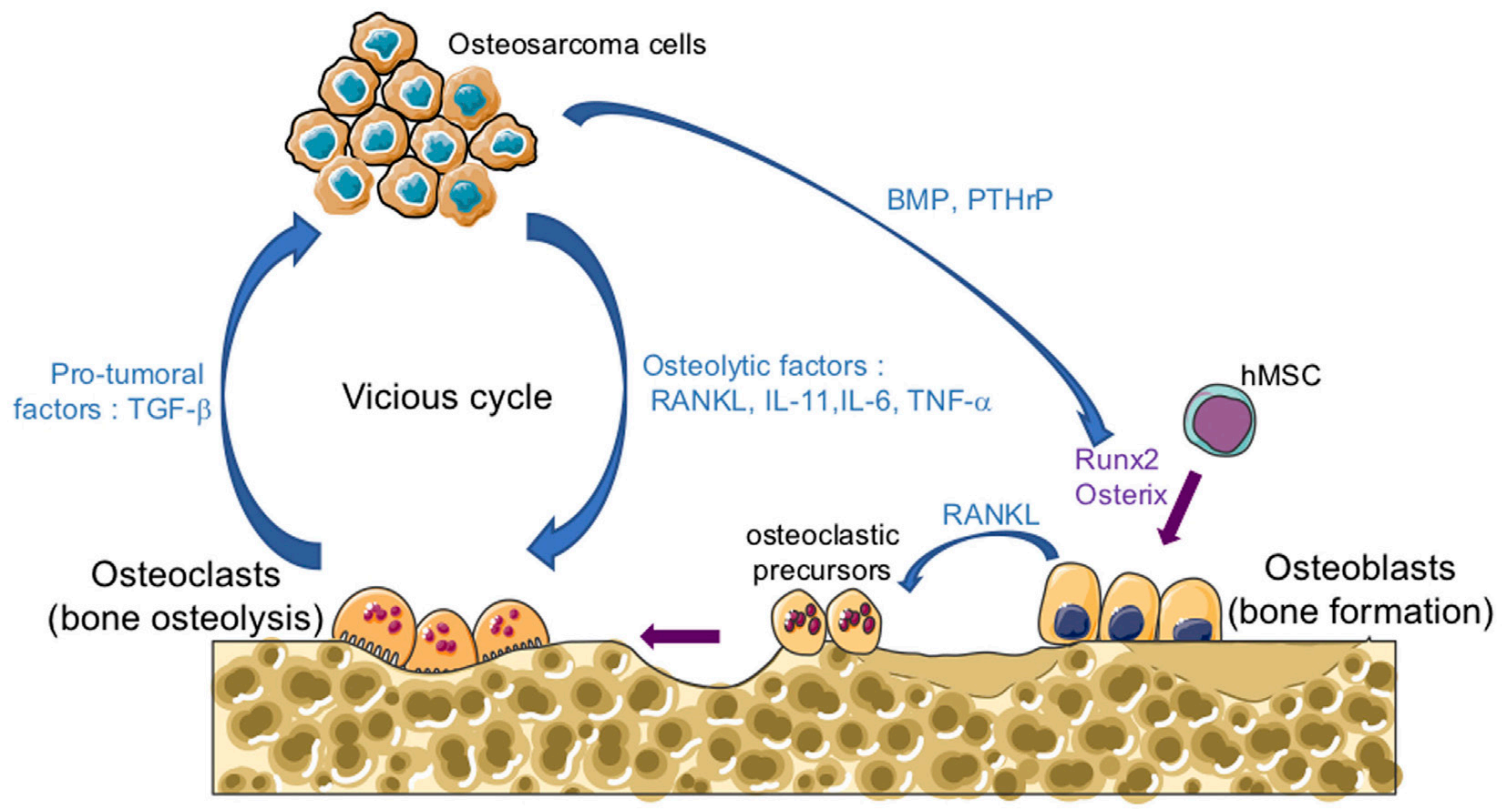
The vicious cycle between tumor and bone cells during osteosarcoma development. Osteosarcoma cells produce soluble osteolytic factors such as receptor activator of nuclear factor kappa-B ligand (RANKL), interleukin-11 (IL-11), IL-6, and tumor necrosis factor-α (TNF-α) that directly activate osteoclastogenesis, leading to bone degradation. Osteosarcoma cells also produce soluble factors, such as bone morphogenetic protein (BMP) or parathyroid hormone-related protein (PTHrP), which stimulate the production of RANKL by osteoblasts and therefore increase osteoclast activity. Osteoblasts are derived from mesenchymal stem cell in response to transcriptional factors such as Runx2 and osterix. Following bone degradation, the growth factors trapped in the bone matrix, such as transforming growth factor-βs (TGF-βs), are released into the bone microenvironment and stimulate both tumor growth and metastatic progression.

One of the main features of osteosarcomas is their influence on bone remodeling as they are characterized by both the formation of osteoid matrix, and by osteolytic lesions. A vicious cyclie between tumor and bone cells occurs during the development of osteosarcoma, promoting tumor growth and metastatic dissemination (Figure 1). In brief, osteosarcoma cells produce soluble osteolytic factors such as interleukin-6 (IL-6), IL-11, tumor necrosis factor-α, or receptor activator of NF-κB ligand (RANKL) that activate osteoclastogenesis, leading to bone degradation. Following this process, growth factors trapped in the bone matrix, such as transforming growth factor-βs (TGF-βs), are released into the bone microenvironment and stimulate tumor growth and metastatic progression (14, 15). The impact of osteoclast activity on osteosarcoma growth and progression has been reported by several studies (16–19). Therefore, therapeutic approaches targeting osteoclasts may be a promising option. Although the use of zoledronate, a strong inhibitor of osteoclast function, in the French randomized OS2006 trial in combination with chemotherapy and surgery, did not show any significant improvement (20), targeting the cytokines released during bone degradation, in particular TGF-β, remains relevant.

## TGF-β Signaling Pathways

In humans, the TGF-β family is composed of 33 members, encoded by 33 different genes, including the TGF-βs, activins, nodal, bone morphogenetic proteins, and growth and differentiation factors (21–23). Of these secreted cytokines, three different isoforms of TGF-βs have been identified in mammals: TGF-β1, -β2, and -β3. TGF-β isoforms are secreted as latent precursor molecules, requiring activation into a mature form for receptor binding. Many activators of latent TGF-βs have been described in the last few decades, including integrins, proteases such as MT1-matrix metalloproteinase (MMP) or others MMPs, and physicochemical factors such as detergents, and ionizing and ultraviolet radiation (22, 24–26). Once activated, TGF-β dimers signal from the membrane to the nucleus by binding to two heteromeric cell surface serine/threonine kinase receptors, named type I (TβRI) and type II (TβRII) receptors. Ligand binding induces the assembly of two TβRI and two TβRII receptors into a heterotetrameric complex in which TβRII phosphorylates a specific serine residue of TβRI and in turn activates the serine/theronine kinase of TβRI (27–29).

**Figure 2:**
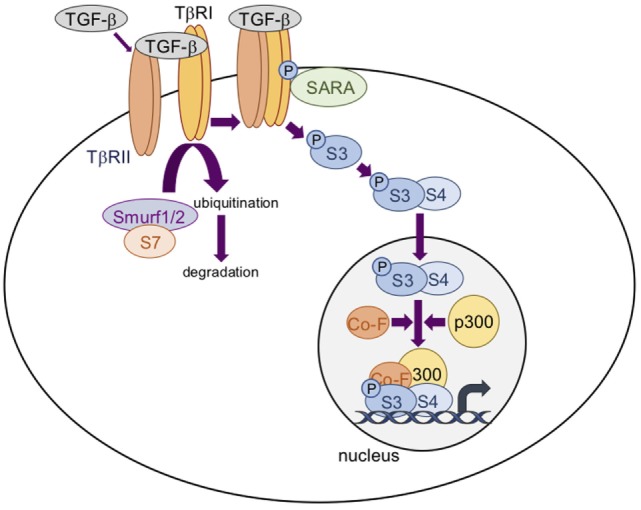
The transforming growth factor-β (TGF-β)/Smad signaling pathway. TGF-β dimers bind to two TβRII receptors that induce the assembly of two TβRI and two TβRII receptors into a heterotetrameric complex in which TβRII phosphorylates and activates TβRI. Smad3 (S3) is then phosphorylated and activated by TβRI. Activated S3 dissociates from the Smad anchor for receptor activation protein (SARA) and recruits Smad4 (S4). This protein complex is translocated into the nucleus to regulate target gene expression in association with cofactors (Co-F) and/or p300. Smad7 (S7) recruits E3-ubiquitin ligases (Smurf1 and Smurf2) to activate TRβI, resulting in receptor degradation.

Transforming growth factor-βs thus activate the Smads cascade (Figure 2), known as the canonical TGF-β signaling pathway. Briefly, receptor-regulated Smads (R-Smads), including Smad2 and Smad3, are phosphorylated and activated by TβRI. Activated R-Smads then dissociate from the Smad anchor for receptor activation protein (30) and recruit the common-mediator Smad (co-Smad), Smad4. This protein complex is translocated into the nucleus to regulate target gene expression. At the regulatory DNA binding sequence of genes, the R-Smad/co-Smad complex activates transcription through physical interaction and functional cooperation of DNA-binding Smads with sequence-specific transcription factors (29). The minimal Smad-binding element contains four base pairs, 5′-AGAC-3′, but binding to other G/C-rich sequences has also been reported (31). Interaction between the R-Smad/co-Smad complex and other transcription factors (either co-activators or co-repressors) generates a high-affinity protein-DNA complex to regulate gene expression. Several inhibitory mechanisms regulate the TGF-β signaling cascade. Of them, Smad7, induced by TGF-β (32), competes with R-Smads for binding to activated TβRI and thus inhibits R-Smad phosphorylation. Smad7 also has the ability to recruit E3-ubiquitin ligases (Smurf1 and Smurf2) to activate TβRI, resulting in receptor degradation (33, 34). Moreover, Smad7 may recruit protein phosphatases to the receptor complex, resulting in its inactivation (35).

In addition to this canonical pathway, TGF-βs are also able to activate Smad independent or non-canonical pathways, including mitogen-activated protein kinases and phosphoInositide3-kinase/AKT (PI3K/AKT) signaling pathways (36). In this context, one of the first non-Smad effectors of the TGF-β receptor complex is TRAF6, implicated in the activation of TGF-β-activated kinase 1, capable of activating the SAP/JNK and p38-kinase pathways (37, 38). More recently, it has been shown that TRAF6 favors the formation of a TβRI/p85α complex, leading to activation of the PI3K/AKT cascade (39).

## TGF-β and Osteosarcoma

**Figure 3:**
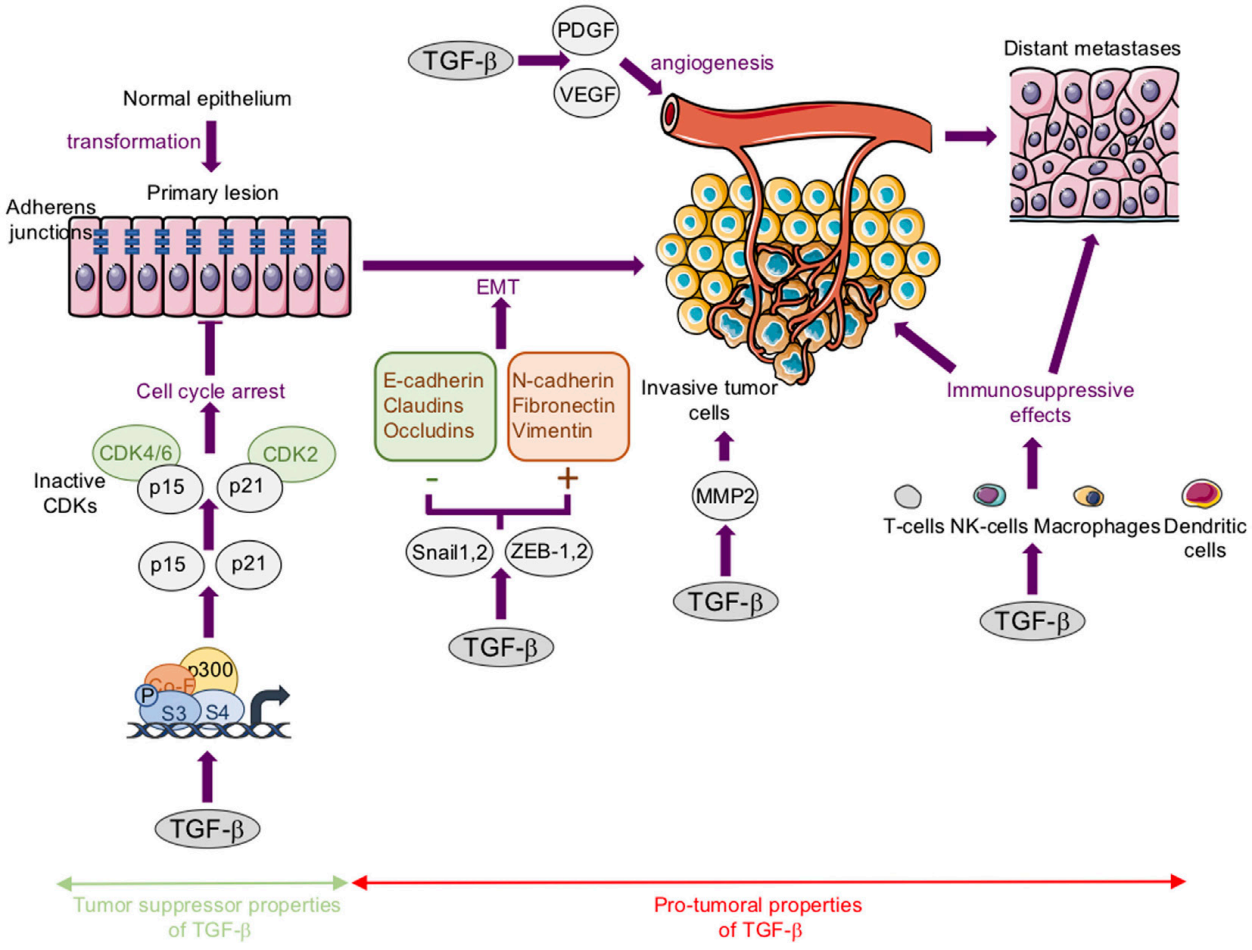
The tumor suppressor and protumoral properties of transforming growth factor-β (TGF-β) in carcinoma. *Tumor suppressor properties*: TGF-βs inhibit cell proliferation largely by inducing the expression of cyclin-dependent kinase (CDK) inhibitors such as p21^Cip1^ (p21) and p15^lnk4b^ (p15). *Protumoral properties*: TGF-βs stimulate epithelial–mesenchymal transition (EMT). This process is associated with a loss or downregulation of E-cadherin, claudins, and occludins, and an upregulation of mesenchymal markers such as N-cadherin, fibronectin, and vimentin. These changes in gene expression are regulated by transcription factors such as Snail-1, Snail-2, ZEB-1, and ZEB-2. TGF-βs stimulate angiogenesis in part by stimulating platelet-derived growth factor (PDGF) and vascular endothelial growth factor (VEGF) expression. TGF-βs favor cancer cell migration and invasion in part by increasing matrix metalloproteinase-2 (MMP) expression. TGF-βs exert immunosuppressive effects *via* the modulation of the activity or biology of immune cells such as T-cells, natural killer cells (NK-cells), macrophages, and dendritic cells.

Regarding carcinoma, it is widely accepted that TGF-βs act both as tumor suppressors in premalignant tumors and as tumor promoters in advanced tumors (15, 40–43) (Figure 3).

Briefly, TGF-β1 acts as a tumor suppressor mainly through its ability to inhibit cell proliferation both by inducing the expression of cyclin-dependent kinase inhibitors such as p21^Cip1^ and p15^lnk4b^, and/or by reducing the expression of proliferative drivers such as c-Myc and cyclin-D (44–46). In this context, alterations or mutations to TGF-β cascade members have been associated with several types of carcinoma (47, 48).

In contrast with carcinoma, it seems that TGF-βs fail to inhibit mesenchymal cell proliferation, particularly in the case of osteosarcoma cells (49, 50) and that TGF-βs exert only protumoral properties in sarcomas through their pro-metastatic effects. In this context, we will focus in this chapter on the pro-metastatic properties of TGF-βs in osteosarcoma. In the last few decades, studies of TGF-βs expression in cancer have correlated TGF-βs levels with the metastatic potential of tumors, suggesting that TGF-βs play a role in tumor progression. In osteosarcoma, TGF-β1 and TGF-β2 expression increase in the sera of patients compared to those of healthy donors (50). This increase in TGF-β production is correlated with high-grade osteosarcoma and associated with the presence of lung metastases (50–52). In addition, our previous results suggest that TGF-β is capable of targeting both tumor cells and their microenvironment. The secretion of TGF-βs by tumor cells or stroma cells can effectively act in an autocrine/paracrine manner to regulate the phenotype and functions of the microenvironment in order to stimulate protumorigenic microenvironmental changes.

### TGF-β Exerts Protumorigenic Functions by Targeting Tumor Cells: TGF-β and Epithelial–Mesenchymal Transition (EMT) or “EMT-Like” Phenomena

The switch in TGF-β properties during carcinogenesis has been associated with the ability of TGF-βs to induce the EMT process (53). This multi-step process, characterized by a decrease in epithelial properties and an increase in mesenchymal ones, promotes the invasiveness of cancer cells and contributes to the development of circulating tumor cells (43, 54). This cellular process involves different molecular and cellular modifications, including a loss of cell-to-cell interactions associated with a loss or downregulation of crucial components in the intercellular junction such as E-cadherin, claudins, occludins, and desmosomes. In parallel, an upregulation of mesenchymal marker expression, such as N-cadherin, fibronectin, and vimentin, is observed. These changes in gene expression are regulated by different transcription factors such as Snail-1, Snail-2 (Slug), ZEB-1, and ZEB-2, or Twist (53, 54). Various secreted factors, such as fibroblast growth factors, hepatocyte growth factor, Wnts, Hedgehog proteins, or TGF-βs, induce EMT or are implicated in EMT (55). The regulation of EMT by TGF-βs has been associated with Smad-dependent and Smad-independent signaling pathways (43, 56). In TGF-β-induced EMT, Smad proteins can induce the expression of transcription factors such as Snail, Slug, and Twist involved in the loss of E-cadherin expression, and in turn in the loss of the E-cadherin adhesion complex (55, 57). Interestingly, several other signaling pathways, such as the Wnt, Hippo, and Sonic Hedgehog cascades, cooperate with the Smad cascade to regulate EMT in many cancer cells (55).

**Figure 4:**
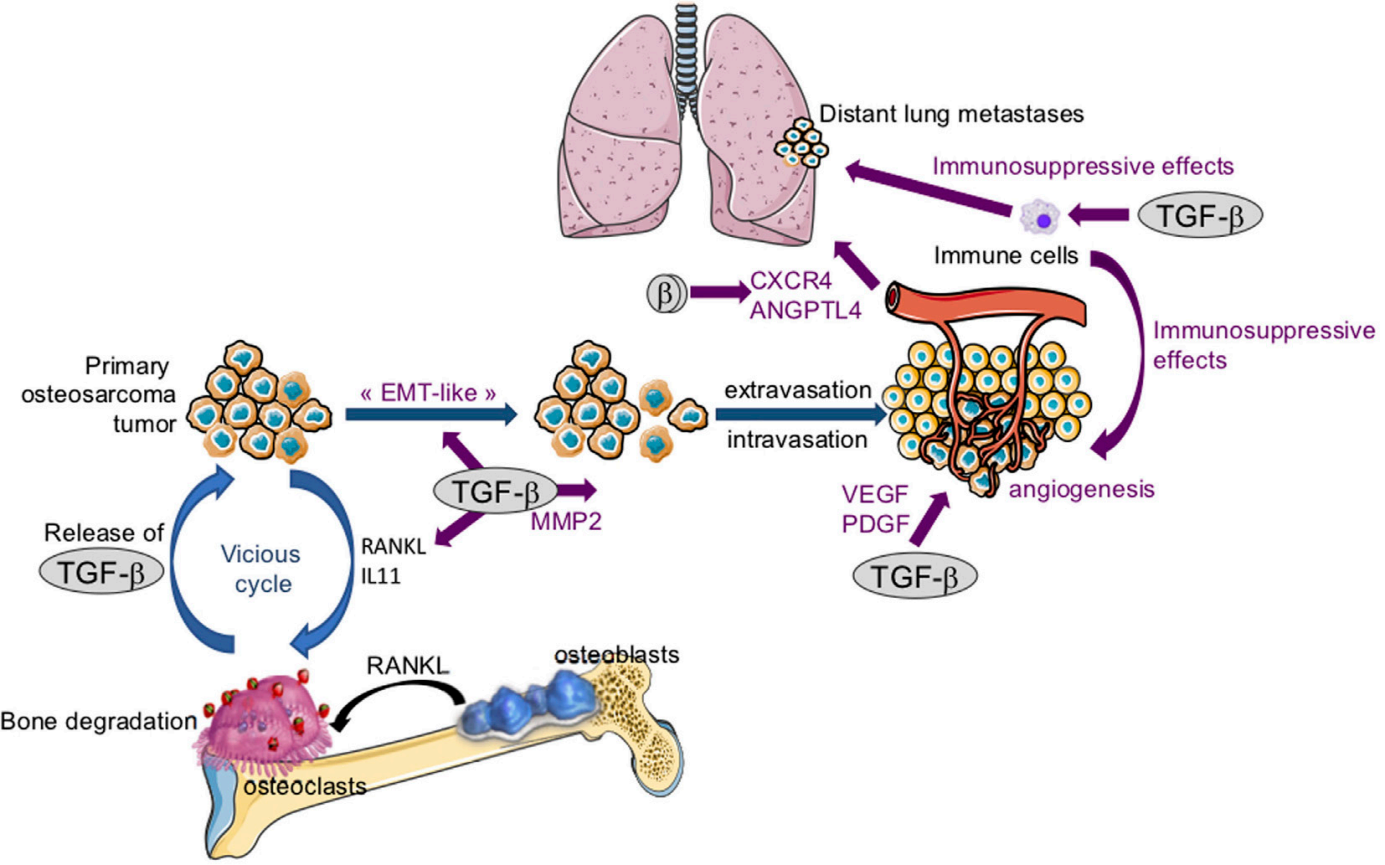
The crucial role of transforming growth factor-βs (TGF-βs) in osteosarcoma tumor growth and metastatic dissemination. Following bone degradation, the TGF-βs trapped in the bone matrix are released and promote osteosarcoma growth and metastatic dissemination by targeting both osteosarcoma tumor cells and their microenvironment. *TGF-βs target osteosarcoma cells*: TGF-βs stimulate “epithelial–mesenchymal transition-like” (EMT), cell migration, and invasion in part by increasing matrix metalloproteinase-2 (MMP-2) expression. *TGF-βs target tumor microenvironment*: TGF-βs upregulate the expression of osteolytic factors such as receptor activator of nuclear factor kappa-B ligand (RANKL) and interleukin-11 (IL-11) and therefore stimulate bone osteolysis and the secretion of protumoral factors. TGF-βs upregulate platelet-derived growth factor (PDGF) and vascular endothelial growth factor (VEGF) expression, and therefore angiogenesis. Finally, TGF-βs exert immunosuppressive effects by regulating immune cell proliferation and activity.

Despite the fact that osteosarcoma arises from transformed cells of mesenchymal origin, numerous studies have demonstrated that an overexpression of EMT-transcription factors such as Snails, ZEBs, or Twist is involved in the pathogenesis of osteosarcoma, making possible an “EMT-like” phenomenon (Figure 4) that promotes the invasive properties of osteosarcoma cells and therefore the formation of metastases at distant secondary sites (58). Osteosarcoma tissues thus exhibit elevated Twist expression compared with non-tumorigenic osteochondroma tissue. In addition, metastatic osteosarcoma (grade III) shows an increase in Twist expression compared with non-metastatic osteosarcoma (grade I/II) (59). In this context, *in vitro* studies have demonstrated that Twist overexpression in SaOS_2_ osteosarcoma cells is associated with both an increase in cell invasive properties and osteosarcoma cell resistance to cisplatin (60). Similarly, Snail-2 is expressed in the three main histological subtypes of long bone osteosarcoma (osteoblastic, chondroblastic, and fibroblastic), and Snail-2 expression is statistically correlated with tumor grade whatever the osteosarcoma subtype (61). Finally, the transcript and protein levels of ZEB-1 are significantly higher in osteosarcoma tissues when compared with normal bone tissues, ZEB-1 levels being increased in patients with lung metastasis (62).

Together, these results demonstrate that an “EMT-like” phenomenon may be associated with the pathogenesis of osteosarcoma. Although the role of TGF-β has not yet been fully defined in this EMT-like process, *in vitro* experiments have demonstrated that the ability of TGF-βs to promote this EMT-like phenomenon (63) may be associated with the pro-migratory effect of TGF-β1 on several osteosarcoma cell lines (50, 64–66). *In vivo* experiments, using molecular (overexpression of the Smad inhibitor, Smad7) and pharmacological (SD-208 and/or halofuginone) approaches, have demonstrated that TGF-βs affect the development of lung metastases in osteosarcoma (50, 67). This effect is associated in large part with the ability of Smad7, SD-208, or halofuginone to block the capacity of TGF-β1 to stimulate osteosarcoma migration and invasion (15, 50, 67).

### TGF-β Exerts Protumorigenic Functions by Targeting the TME: Angiogenesis, Bone Remodeling, and Immunosurveillance

Tumors are heterogeneous tissues in which tumor cells are surrounded by and interact with a complex TME, composed of both cellular and non-cellular components. This TME plays a critical role in determining the fate of tumor cells during tumorigenesis and metastasis. Aberrant upregulation of TGF-β expression in the TME has thus been implicated in promoting cancer progression and metastasis (68–70). In this context, we will focus the following chapter on the role of TGF-β in tumor angiogenesis, bone remodeling, and modulation of the immune system.

#### TGF-β and Tumor Angiogenesis

Angiogenesis is a complex biological process that plays a crucial role in sustaining the microenvironment, growth, and metastatic potential of several tumors (71). Schematically, this process favors the formation of blood vessels, increasing the supply of nutriments and providing an entry point for the invasive cells (72). The proliferation, migration, and maturation of endothelial cells are critical steps involved in regulating the angiogenic process. A crucial primary cytokine that drives this process is the secreted vascular endothelial growth factor (VEGF) which stimulates the proliferation and migration of endothelial cells, and the formation of vessels (73–75). Other secreted cytokines, such as platelet-derived growth factor (PDGF), also play a major role in blood vessel formation by inducing vessel maturation mainly by increasing pericyte migration and the induction of pericytes coverage (70, 76). Regarding the role of TGF-β in angiogenesis, TGF-β1 KO mice display a phenotype that is defective in angiogenesis (77, 78). In addition, the loss of endothelial type I or type II TGF-β receptors in mice results in a decrease in vessel formation (79, 80). With regard to tumor angiogenesis, high circulating levels of TGF-β1 are correlated with increased tumor angiogenesis in many forms of cancer (41). Different experimental models for tumor progression, as well as data from human biopsies, have thus demonstrated that a high expression of TGF-β is associated with the expression of angiogenic factors (81) and correlates with the increase in new vessel formation (82, 83). Interestingly, TGF-β1 is able to increase VEGF (84) or PDGF (85) expression in many cancer cells, and therefore to induce tumor angiogenesis (86, 87).

For osteosarcoma pathogenesis (Figure 4), VEGF expression has been associated with microvascular density (88), and patients with high VEGF expression levels exhibit lower disease-free survival (89). *In vitro* studies revealed that U2OS osteosarcoma cells secrete a PDGF-like growth factor (90), and a malignancy-dependent co-expression of PDGF and PDGF receptors has been observed in the biopsies of osteosarcoma patients (91). Finally, *in vivo* experiments demonstrated that blocking TGF-β signaling by Smad7 overexpression in osteosarcoma cells or treating mice with the ALK5 inhibitor SD-208, reduces expression of both the endothelial marker CD146 and PDGF (50).

#### TGF-β and Bone Remodeling

Two cell lineages, the mesenchymal osteoblastic and hematopoietic osteoclastic lineages, are implicated in bone remodeling. Schematically, the osteoblasts and osteoclasts drive bone formation and resorption, respectively. The three mammalian isoforms of TGF-β (TGF-β1, -β2, and -β3) are found in bone (92). The role of these TGF-βs in skeleton development in general, and specifically during bone remodeling, is complex. *In vitro*, TGF-β1 stimulates the proliferation and migration of mesenchymal stem cells during the early stages of osteoblastogenesis, and inhibits both the differentiation of mesenchymal stem cells into osteoblasts, and the activity of osteoblasts in the late stages of osteoblastogenesis (15, 93–95). For osteoclastogenesis, TGF-β1 affects bone resorption in a dose-dependent manner (15, 92). *In vitro*, low doses of TGF-β1 stimulate the differentiation of osteoclasts, and high doses of TGF-β1 inhibit the differentiation of osteoclasts through modulation of RANKL and osteoprotegerin expression by osteoblasts (96). As a consequence, *in vivo* experiments indicate that TGF-βs favor bone resorption and destruction (15).

Interestingly, blocking the TGF-β signaling pathway in osteosarcoma cells reduces the bone osteolysis associated with tumor growth and, in turn, tumor progression. Indeed, in a xenograft murine model of osteosarcoma using human HOS or SaOS_2_ cells, Smad7 overexpression in tumor cells inhibited the tumor-associated bone destruction by both promoting ectopic bone formation and preventing trabecular bone osteolysis (50). One hypothesis to explain this phenomenon is that blocking the TGF-β cascade in osteosarcoma cells inhibits the expression and secretion of the TGF-β target genes, such as RANKL and IL-11, which stimulate osteoclast activity (50).

One of the hallmarks of the extra cellular matrix in tumor progression is also upregulation of proteolytic enzymes such as MMPs (68, 69, 97). For osteosarcoma, several studies have shown that highly invasive osteosarcomas express higher levels of MMP-2 than weakly invasive tumors, and osteosarcoma cell invasion is associated with MMP-2 expression (98, 99). In addition, MMP-2 expression correlated with prognosis and response to chemotherapy (100, 101). Interestingly, blocking the TGF-β signaling pathway in osteosarcoma cells reduces the formation of lung metastases, mainly by inducing a decrease in MMP-2 expression in tumors. In addition, blocking the TGF-β cascade in tumor cells inhibits the expression and activation of MMP-2 and the ability of TGF-β to stimulate osteosarcoma cell migration and invasion (50) (Figure 4).

#### TGF-β and the Immune System

Transforming growth factor-βs are secreted cytokines that have multiple immunosuppressive properties (102) *via* modulation of the activity or biology of several cells in the immune system, such as T-cells, natural killer (NK) cells, macrophages, and dendritic cells (41, 103, 104).

These immunosuppressive abilities of TGF-βs include inhibition of T-cell proliferation, inhibition of T-cell differentiation into cytotoxic T lymphocytes and helper T cells, and inhibition of the T-cell stimulatory functions of antigen-presenting cells (104). For example, in a mouse model expressing a dominant negative form of TβRII restricted to CD4^+^ and CD8^+^ T-cells, an antitumor response is observed against melanoma progression (105).

The functional activation of NK cells, which play a crucial role in the antitumor response by recognizing and destroying tumor cells, is inhibited by TGF-β *via* different mechanisms (105). For example, TGF-βs antagonize the IL-15-induced cell proliferation associated with NK-cell activation and thus block the functional activation of NK-cells (106). The biological functions of the DC cells involved in the activation of the immune response, and therefore in determining the host response to primary tumor cells, are also regulated by TGF-β (104). For example, TGF-β increases the expression of inhibitor of differentiation 1 driving the switch from dendritic cell differentiation to myeloid-derived suppressor cell expansion during tumor progression (107).

Solid tumors are usually invaded by macrophages called TAMs. These TAMs are classically divided into two categories: M1 polarized macrophages, identified as antiumor cells, and M2 polarized macrophages, identified as protumor cells (108, 109). In this context, certain studies have demonstrated the ability of TGF-βs to drive the induction of macrophage polarization from M1 to M2 subtypes (110). All these immunosuppressive properties of TGF-βs induce tumor evasion from immune response (41, 68, 103, 104).

The immune environment of osteosarcoma is mainly composed of myeloid cells (monocytes, macrophages, and dendritic cells) and T-lymphocytes (111). Osteosarcoma cells control the recruitment and differentiation of immune-infiltrating cells, and establish a local immune tolerant microenvironment, allowing the tumor to grow (111).

While initial studies demonstrated that macrophages are associated with reduced metastasis and improved survival in high-grade osteosarcoma (112), recent studies have shown that TAMs are associated with better overall survival (113, 114). T-cells are the other cell population represented in the immune infiltrate in osteosarcoma (111). Recent studies have indicated that the CD8^+^/FOXP3^+^-ratio is a strong prognostic factor for osteosarcoma at diagnosis (115).

## Conclusion and Clinical Relevance

In carcinoma, TGF-βs exhibit both tumor suppressor and protumoral properties, depending on the stage of the disease. In this context, it seems that the timing of therapies targeting TGF-βs needs to be considered with great precision. For sarcoma, and specifically osteosarcoma, TGF-βs mainly seem to exert protumoral properties by targeting both tumor cells and their microenvironment. It therefore appears that TGF-βs, major drivers for osteosarcoma, could be considered as promising therapeutic targets in this disease. In the last decade, different strategies targeting TGF-βs have been developed (Table 1), including anti-ligand antisense oligonucleotides, which are capable of binding human TGF-β2 mRNA (trabedersen), antibodies that target ligands or receptors, such as fresolimumab, a humanized mAB against TGF-β, and drugs against TGF-β receptor kinases, such as galunisertib (LY2157299, a TβRI inhibitor) [reviewed in Ref. (116–118)].

**Table 1:** Transforming growth factor-β (TGF-β) inhibitors in clinical development in cancer (ClinicalTrials.gov).

Drug	Targets	Trial number	Cancer	Clinical development phase
**TGF-β ligand inhibitors**				

Fresolimumab (GC-10008)	panTGF-β	NCT00356460	Advanced Renal Cell Carcinoma or Malignant Melanoma	I
		NCT00923169	Advanced Renal Cell Carcinoma or Malignant Melanoma	I
		NCT01472731	Relapsed Malignant Glioma	II
		NCT01112293	Relapsed Malignant Pleural Mesothelioma	II
		NCT01401062	Metastatic Breast Cancer	II
		NCT02581787	Early Stage Non-small Cell Lung Cancer	I, II

Trabedersen (AP12009)	TGF-β2	NCT00844064	Pancreatic Neoplasms, Melanoma or Colorectal Neoplasms	I
		NCT00431561	Glioblastoma or Anaplastic Astrocytoma	II
		NCT00761280	Anaplastic Astrocytoma or Glioblastoma	III

Belagenpumatucel-L (Lucanix)	TGF-β2	NCT00676507	Advanced Non-small Cell Lung Cancer	III
		NCT01058785	Lung Neoplasm, Carcinoma, Bronchogenic	II
		NCT01279798	Lung Neoplasm or Advanced Carcinoma Non-Small Cell Lung Cancer	III

Recombinant human granulocyte macrophage-colony stimulating factor (rhGMCSF)/shRNAfurin vaccine	TGF-β1,2	NCT01309230	Ovarian Cancer	II
		NCT01505166	Colon Cancer	II
		NCT01867086	Ovarian Cancer	II
		NCT01551745	Ovarian Cancer	II
		NCT01061840	Ewings Sarcoma, Non Small Cell Lung Cancer, Liver Cancer	I
		NCT01453361	Advanced Melanoma	II

**TGF-β receptor inhibitors**				

Galunisertib (LY2157299)	TβRI	NCT02734160	Metastatic Pancreatic cancer	I
		NCT02154646	Pancreatic Neoplasm	I
		NCT01722825	Advanced or Mestastatic Neoplasm	I
		NCT02452008	Prostate Cancer	II
		NCT02538471	Metastaric Breast Cancer	II
		NCT02423343	Refractory or Recurrent Non-Small Cell Lung Cancer Recurrent, or Hepatocellular Carcinoma	I, II
		NCT02304419	Neoplasm	I
		NCT01373164	Pancreatic Cancer	I, II
		NCT02672475	Triple-Negative Breast Carcinoma	I
		NCT02688712	Rectal Adenocarcinoma	II
		NCT02240433	Hepatocellular Carcinoma	I
		NCT03206177	Carcinosarcoma of the Uterus or Ovary	I
		NCT02906397	Advanced Hepatocellular Carcinoma	I
		NCT01220271	Glioma	I, II
		NCT01582269	Recurrent Glioblastoma	II
		NCT01246986	Hepatocellular Carcinoma	II
		NCT01682187	Glioma	I
		NCT02178358	Hepatocellular Carcinoma	II
		NCT02734160	Metastatic Pancreatic Cancer	I

TEW-7197	TβRI	NCT02160106	Advanced Stage Solid Tumors	I
		NCT03143985	Multiple Myeloma	I

PF-03446962	ALK1	NCT02116894	Colorectal Cancer	I
		NCT00557856	Advanced Solid Tumors	I
		NCT01620970	Transitional Cell Carcinoma of Bladder	II
		NCT01337050	Stomach Cancer	I
		NCT01911273	Advanced or Metastatic Liver Cancer	II
		NCT01486368	Malignant Pleural Mesothelioma	II

IMC-TR1	TβRII	NCT01646203	Advanced Solid Tumors	I

*Fresolimumab (GC1008) is an antibody capable of neutralizing TGF-β1,2,3. Trabedersen (AP 12009) is an antisense oligodeoxynucleotide specific to human TGF-β2. Belagenpumatucel-L (Lucanix) is a TGF-β2 antisense transgene. rhGMCSF/shRNAfurin vaccine is a dual-modulatory autologous whole cell vaccine (bi-shRNA furin and GMCSF autologous tumor cell vaccine), incorporating the rhGMCSF transgene and the bifunctional shRNA-furin to block pro-protein conversion to active TGF-β1,2. Galunisertib (LY2157299) is a TβRI inhibitor. TEW-7197 is an inhibitor of the protein serine/threonine kinase activity of TβRI. PF-03446962 is a monoclonal antibody against ALK1. IMC-TR1 is a monoclonal antibody against TβRII*.

With these strategies, certain trials have shown positive results. For example, trabedersen has been successfully tested in patients with recurrent or refractory high-grade glioma. This randomized, open-label, dose-finding phase IIb study evaluated the efficacy of trabedersen administered intratumorally at doses of 10 or 80 μM compared with standard chemotherapy (temozolomide or procarbazine/lomustine/vincristine) in patients with recurrent/refractory glioblastoma multiform or anaplastic astrocytoma (AA). Analysis of the AA subgroup revealed a significant benefit regarding the 14-month tumor control rate for 10 μM trabedersen vs chemotherapy. In addition, the trend for the 2-year survival rate was for the superiority of 10 μM trabedersen vs chemotherapy (119). Concerning galunisertib, the clinical benefit was observed in 12 of the 56 patients with glioma (21.4%) (120). Most other clinical trials were negative or are still in progress. It should be noted that some drugs that block TGF-β activity have shown cardiotoxicity as side effects.

However, these strategies that target the TGF-β pathway could be considered and proposed in the therapeutic arsenal for osteosarcoma patients.

## Author Contributions

All authors listed have made a substantial, direct, and intellectual contribution to the work and approved it for publication.

## Conflict of Interest Statement

The authors declare that the research was conducted in the absence of any commercial or financial relationships that could be construed as a potential conflict of interest.
